# Transactions of the Virginia Society of Surgeon Dentists, at Their Third Annual Meeting

**Published:** 1845-12

**Authors:** 


### ARTICLE X.

*Transactions of "The Virginia Society of Surgeon Dentists," at their Third Annual Meeting*, begun and held in the Office of Dr. Samuel Lethbridge, in the City of Richmond, on the second Wednesday in October, 1845.

The President took the chair, and called the Society to order at 12 o'clock, M.

The Secretary called the roll of members, and read the excuses of absentees. A constitutional quorum being present, the Society proceeded regularly to business.

The Committee appointed at the last annual meeting to procure from the state legislature an act of incorporation, reported that they had been successful, and laid before the Society the following charter of incorporation:

### AN ACT

*Incorporating the Virginia Society of Surgeon Dentists.*

(Passed February 3d, 1845.)

Whereas, Samuel Lethbridge, John G. Wayt, James D. M'Cabe, and others, citizens of the State of Virginia, have formed themselves into a society, under the title of "The Virginia Society of Surgeon Dentists," whose objects are the promotion of dental science, the establishment of a library and cabinet of anatomical preparations, for the purpose of introducing a sound system of dental education: and it having been represented to the General Assembly, that the members of said society are desirous of obtaining a charter of incorporation, that they may, with lawful protection, carry out their laudable purposes.

*Be it therefore enacted,* That the members of the aforesaid Society, together with such others as shall hereafter be associated with them, and their successors, are hereby created, a body politic and corporate, by the name and style of the "Virginia Society of Surgeon Dentists," and by that name shall have perpetual succession, and a common seal, and may hold and dispose of lands and tenements, goods and chattels, "not exceeding twenty-five thousand dollars at any one time," and may sue and be sued, plead and be impleaded.

The said society may also enact such constitution and by-laws, ("not repugnant to the laws of this state, and of the United States,") as they may think proper, for carrying into effect the object of the institution. And may do all other acts, proper for corporate bodies to do.

The legislature reserves the right to modify or repeal this act.

This act shall be in force from the passing thereof.

I certify that the foregoing is a true copy.

GEORGE W. MUNFORD, C. H. D.

*February* 7, 1845.

On motion of Dr. W. W. H. Thackston,

*Resolved*, That this Society does hereby accept the charter granted by the legislature, and that we proceed to organize under its sanction and authority.

On motion of Dr. M'Cabe, seconded by Dr. Thackston, the Society proceeded to organize under the charter, by the adoption of the following Constitution and By-laws:

### CONSTITUTION.

### PREAMBLE.

Whereas, the abuses, and flagrant malpractices perpetrated by individuals engaged in the practice of "Dental Surgery," have long been sources of unmitigated obloquy and contempt—visited alike upon the scientific and upright practitioner of our art, and the "gasconading charlatan;" and conceiving that the abuses in question may be partially, if not wholly corrected; that the imposture which has so long degraded the dental profession in *Virginia*, may, to a considerable extent, be suppressed—the interest of the science promoted, its character elevated, and a free and open interchange of sentiment and opinion, upon the various subjects of interest which appertain to our profession, *be established;* we whose names are hereunto affixed, have organized ourselves into a Society, which shall be known and designated by the name and title of "THE VIRGINIA SOCIETY OF SURGEON DENTISTS."

ARTICLE I.—*Of Officers.*

*Sec.* 1. The officers of this Society shall consist of a President and Vice-President, a Recording and Corresponding Secretary, an Executive, Examining and Publishing Committee.

*Sec.* 2. The election of the above named officers shall be by ballot, at a regular annual meeting of the Society, a majority of votes determining the election.

ARTICLE II.—*Of Members.*

There shall be two classes of members, known and recognized as Acting Members or Fellows, and Honorary Members; the former consisting of those who subscribe to this constitution, either personally or by proxy, and pay into the treasury the annual dues required by this constitution, and the latter embracing such members as are merely elected to membership.

ARTICLE III.—*Requisitions for Membership.*

*Sec.* 1. Each and every acting member of this Society, shall have been such by virtue of his attendance in person, by proxy, or letter, at the time of its formation, or shall afterwards be elected as prescribed by this constitution, and subscribe to the same.

*Sec.* 2. Each and every acting member shall pay into the treasury of the Society, the annual sum of three dollars, for the benefit of its funds.

*Sec.* 3. Each and every acting member shall be required to attend the Society at least once in *two* years, unless excused for satisfactory reasons.

ARTICLE IV.—*Election of Members.*

All members of the Society, excepting those who were acting members, at the time of its organization, and the adoption of this constitution, shall be elected as follows, viz. The candidate for membership shall be proposed at a regular meeting by the Executive Committee, whereupon two tellers shall be appointed by the presiding officer, who shall collect the ballots, consisting of slips of paper, having legibly written on them either *yea* or *nay.* In case two-thirds of the members present vote in the affirmative, the candidate shall be declared to have been duly elected a member of this Society.

ARTICLE V.—*Expulsion of Members.*

Any member of the Society may be expelled for immoral conduct, malpractice, or other sufficient cause, on motion of one member, seconded by another, and sustained by a majority of two-thirds of the members present, at any regular meeting of the Society.

ARTICLE VI.—*Of Meetings of the Society.*

The meetings of the Society shall be held annually, by adjournment from time to time, and from place to place, agreeably to the will of the Society.

ARTICLE VII.—*Of the Resources of the Society.*

*Sec.* 1. This Society may receive contributions of money, books, or other property, which may be either sold in aid of its purposes, or otherwise used.

*Sec.* 2. Each and every candidate examined and admitted to membership, shall pay into the treasury a fee of five dollars, before receiving his *Diploma* or *Certificate.*

*Sec.* 3. Each and every honorary member, who shall become so by election, shall pay ten dollars for the benefit of its funds, which shall entitle him to a diploma or certificate of membership.

ARTICLE VIII.—*Disposition of the Funds.*

The funds of this Society may be appropriated at any time, in that way and manner, and for such purposes as the Society in its wisdom may deem expedient or necessary.

ARTICLE IX.—*Of the Distribution of the Effects of the Society in the event of its Dissolution.*

*Sec.* 1. In case a dissolution of this Society shall at any time be proposed, a meeting shall be called specially for that purpose, and the consent of three-fourths of its members be required to effect it.

*Sec.* 2. Should the Society be dissolved by its own act, the property belonging to it shall be sold by order of the President, or by any three fellows, who shall have authority from a majority of subscribing members, and the assets of the sale shall be equally divided among the above mentioned members.

ARTICLE X.—*Of the Qualifications of Candidates who had not entered upon Professional Practice.*

*Sec.* 1. The candidate shall be at least twenty-one years of age, shall have a good English education, shall exhibit evidence of unexceptionable *moral character*, and shall have studied and practised for the full term of two years with some practical dentist, known as such to this Society.

*Sec.* 2. No candidate for membership who can exhibit a diploma from any dental college, regularly chartered in any of the United States, shall be subject to an examination by the Examining Committee of this Society; but shall be entitled to his diploma, by becoming a member and subscribing to the consti tution, and complying with the by-laws.

ARTICLE XI.—*Of the Quorum, and Contingency of there being no Quorum.*

*Sec.* 1. Five acting members or fellows of this Society, shall, besides the President, be necessary for the transaction of any business at the opening of any annual meeting.

*Sec.* 2. In the event of a failure to procure a quorum at any regular meeting of the Society, the time of meeting on the following year shall be the time and the place, as on the preceding year.

ARTICLE XII.—*Of Alterations, Amendments, &c. of the Constitution.*

Any alteration, amendment, or revision of this constitution, may be made at any regular meeting of the Society, by joint assent of three-fourths of the fellows present at such meeting.

### BY-LAWS.

ARTICLE I.—*Of the Duties of Officers.*

*Sec.* 1. It shall be the duty of the President to preside at all meetings of the Society, when present; to sign the diplomas conferred on members during his term of office; to confer all honorary distinctions awarded by the Society, whether degrees of dental surgery, medals or other testimonials; and to draw upon the treasurer for all moneys appropriated by the Society.

*Sec.* 2. It shall be the duty of the Vice-President to preside at all meetings of the Society in the absence of the President, and to perform the other prescribed duties of the President, in case of his death, resignation, sickness, or other disability.

*Sec.* 3. A President *pro tem.* shall be appointed to preside at any meeting, when the President and Vice-President are both absent.

*Sec.* 4. It shall be the duty of the Recording Secretary, to note in a book kept for the purpose, all the proceedings of the Society, during the term of his office, and to furnish copies of any parts thereof, at the order of the President, or his substitutes; and, also, to deliver the said book and proceedings to his successor in office. He shall also keep the seal of the Society, together with the copper-plate, from which diplomas, certificates, or degrees, are struck; also all the records belonging to the Society, designed to be preserved; and he shall furthermore countersign all diplomas, and set the seal of the Society to all instruments requiring the same; he shall also notify every member of the time and place of meeting of the Society, at least two months before the time of said meeting.

*Sec.* 5. It shall be the duty of the Corresponding Secretary, to conduct the domestic and foreign correspondence of the Society, in accordance with the advice of the President, or the express will of the Society communicated to him by the Recording Secretary, and at his own discretion, in cases of emergency, provided it does not interfere with any of the prescribed rules of the Society.

*Sec.* 6. It shall be the duty of the Treasurer to keep all the moneys of the Society committed to his trust; to pay them over to the order of the President, countersigned by the Secretary, and to keep a correct account of the same in a book or books kept for that purpose.

*Sec.* 7. It shall be the duty of the Librarian to keep the books belonging to the Society's library; to obtain insurance on the same; and to take receipts of all persons who receive books from his hands by order of the Society; and in case of failure of the members receiving such book, to return it in twelve months, he shall forfeit and pay to the Society four times its price.

*Sec.* 8. It shall be the duty of the Executive, Examining and Publishing Committees, to examine the accounts of the Treasurer, and report the same to the Society at its annual meetings; to see that all the rules and regulations of the Society are duly enforced and executed; and it shall also be the duty of such committee, to inquire into the professional reputation and character of all candidates proposed, or who may apply for membership, and report the same to the Society, which, being satisfactory, such candidates shall be balloted for, for admission into the same. It shall also be their duty to appoint a time and place, at least once in each year, for the examination of applicants for the degree of doctor of dental surgery; to examine such persons strictly and faithfully, in relation to their qualifications to enter upon the practice of the profession; and their certificate shall entitle the holder to receive the diploma of the Society conferring the degree of doctor of dental surgery. It shall also be their duty to superintend the printing and publishing of all books, tracts and other documents, issued by the Society.

ARTICLE II.—*Of the Emoluments of Office.*

No officer shall receive any emolument unless in cases of special appropriation, excepting the Recording Secretary, whose duty it shall be to attend the meeting of the Society next subsequent to his election; and who shall be entitled to his travelling expenses and the sum of five dollars per diem during his necessary absence from business, in all cases where he is compelled to leave his place of residence.

ARTICLE III.—*Of the Powers Vested in the Vice-President.*

The Vice-President may, if there be a sufficient number of the members of this Society residing in his immediate vicinity, convene them at any time, and when so convened, they shall have power to elect such officers from among their own number as may be necessary for the transaction of whatever business of a local nature may come before them; and it shall be obligatory on them to report their proceedings regularly at each annual meeting of the Society.

ARTICLE IV.—*Of the Privileges of Members.*

*Sec.* 1. Each and every acting member, or fellow of the Society, shall be entitled to debate and vote on all questions agitated at any meeting thereof, agreeably to the by-laws and standing rules and regulations of the Society; and he shall moreover be eligible to any office in the gift of the Society, and, furthermore, he shall be entitled to a diploma, or degree of doctor of dental surgery.

*Sec.* 2. Each and every honorary member shall be entitled to a seat at all meetings of the Society; shall have the privilege of debating all questions not involving pecuniary expenditure; and shall receive the diploma, or degree of doctor of dental surgery, by paying therefor to the Treasurer of the Society the sum of ten dollars.

ARTICLE V.—*Of the Appointment of Members to Certain Duties.*

*Sec.* 1. It shall be the business of the Society, at each annual meeting, to appoint five of its members to prepare dissertations on some subjects connected with the profession; and also one member to deliver an opening address at the subsequent meeting.

*Sec.* 2. It shall moreover be the duty of the Society, from time to time, to appoint certain individuals to prepare essays or any other documents ordered by the Society.

ARTICLE VI.—*Of Awarding Premiums.*

*Sec.* 1. This Society may award premiums to members, for dissertations on subjects that may at any time be specified by the Society at an annual meeting; also, for important improvements in mechanical dentistry, and the manufacture of incorruptible teeth; and also for any other suitable object.

*Sec.* 2. The question of preference in all such cases shall be determined by the judgment of a special committee, appointed by the Society.

*Sec.* 3. Unsuccessful dissertations shall be at the disposal of their respective authors.

ARTICLE VII.

*Sec.* 1. No member shall speak more than twice to any question, without express permission of the Society, and then only fifteen minutes at each time.

*Sec.* 2. Any member shall be liable to be called to order for personal recrimination of any other member, or for wandering in his remarks from the subject of debate.

ARTICLE VIII.

These by-laws may be altered or amended at any annual meeting of this Society, by a vote of two-thirds of the members present.

RESOLUTIONS HAVING THE ACTION OF BY-LAWS.

*Resolved,* That the members of this Society be requested to furnish reports, in writing, at each annual meeting, of all interesting and anomalous cases occurring in their practice during each year.—*Adopted session of* 1844.

*Resolved,* That hereafter, candidates for examination before the committee of this Society, shall be required to present a written *thesis* on some subject selected by themselves, unless excused for good reasons, by the committee.—*Adopted session of* 1844.

*Resolved,* That such members as fail to pay their annual dues for three years successively, thereby forfeit their membership; and the Secretary is hereby instructed to report them to the Society on the occurrence of such delinquency.—*Adopted session of* 1845.

*Resolved,* By the Virginia Society of Surgeon Dentists, that we believe the use of all *pastes* and *cements*, of which mercury is a part, entirely unfit for, and highly objectionable as fillings for carious teeth—that the use of them in dental practice is empirical, and is hereby declared to be MALPRACTICE.—*Adopted session of* 1845.

On motion, *Ordered,* That the officers elected at the last annual meeting be authorized to discharge their various duties until a new election is had.

There being a vacancy in the Executive, Examining and Publishing Committee, occasioned by the absence of Drs. Murrell and Pierce, the President appointed Drs. S. M. Sheppard and W. C. Crump to fill the vacancy.

The Executive, Examining and Publishing Committee reported the amount expended by the Secretary for printing to be $13 75, which was by them approved.

On motion, *Ordered,* That the report be received and that the amount of expenditure be paid by the Treasurer.

The period for the reception of essays having arrived, they were called for, and the call responded to by the several gentlemen previously appointed.

Dr. W. C. Crump described a case of full dentition at birth, seen by him while on a recent visit to North Carolina. The subject was a still-born black child. The dental arches exhibited the full deciduous denture; they were of a cartilaginous structure, with appearance of imperfect ossification on the anterior surfaces.

Dr. Lethbridge gave an interesting description of an anomalous dentition. The subject a child, resident in Orange county, Virginia.

Dr. W. W. H. Thackston gave a highly interesting history of a singular case of decomposition of the earthy portion of the crown of an inferior dens sapientiæ. The tooth had caused severe pain; he was called upon to extract it; he found the crown about three-fourths covered by the gum. On attempting its removal, the whole crown and neck came way, leaving the fangs. The crown presented the following appearances: There was no cavity—the color dark brown; the consistence similar in all respects to an *osteo sarcoma;* that is, cartilaginous, and interspersed with patches of bone; the fangs were perfectly formed, and healthy. The doctor promised a more full report in a few days.

Several gentlemen gave interesting descriptions of various forms of dental disease, and mode of treatment adopted.

Drs. M'Connell, Hamlin, Sheppard and M'Cabe participated in the discussions and exemplifications.

On motion, the Society took a recess until 7 o'clock, P. M.

### EVENING SESSION.

The Society assembled pursuant to adjournment at 7 o'clock, P. M. The President in the chair.

Dr. W. W. H. Thackston nominated Mr. Abner D. Lewis for membership, and recommended him as a young gentleman of studious habits and high moral character; he was eligible by the constitution, and asked to be examined. His application was referred to the Examining Committee.

The Treasurer presented his report, which being audited by the regular committee, was approved and ordered to be filed with the papers of the Society.

The Examining Committee reported the name of Mr. Abner D. Lewis, who had presented a specimen of his artificial work, and had been rigidly examined on the theory and practice of dental surgery, and having found him qualified to give advice and practice that profession, they recommended him to membership and to the honors of the Society. The ballot having been taken and found unanimous in his favor, the President declared him a member of the Society and entitled to its diploma; which was accordingly granted to him.

On motion of Dr. M'Connell,

*Resolved,* That such members of this Society as fail to pay their annual dues for three years successively, thereby forfeit their membership; and the Secretary is hereby instructed to report them to the Society upon the occurrence of such delinquency.

On motion, the Society proceeded to the election of officers for the ensuing twelve months, whereupon the following gentlemen were declared duly elected:

S. LETHBRIDGE, D. D. S., PRESIDENT.
JOHN G. WAYT, D. D. S., *Vice-President.*
JAMES D. M'CABE, D. D. S., *Cor. & Rec. Sec'y.*
S. M. SHEPPARD, D. D. S., *Treasurer.*

| | |
|---|---|
| W. W. H. THACKSTON, D. D. S.<br>JOHN M'CONNELL, D. D. S.<br>T. B. HAMLIN, D. D. S.<br>J. W. SHEPPARD, D. D. S.<br>W. C. CRUMP, D. D. S. | *Executive, Examining and Publishing Committee.* |

Dr. W. W. H. Thackston was appointed to deliver the address at the next annual meeting, in October, 1846.

The following gentlemen were appointed to prepare Essays to be read before the Society at the annual meeting in 1846:— Drs. Abner D. Lewis, S. M. Sheppard, William C. Crump, T. B. Hamlin and J. D. M'Cabe.

On motion, *Ordered,* That when this Society adjourn it will adjourn to meet in the town of Petersburg on the second Wednesday in October, 1846.

On motion of Dr. James D. M'Cabe, seconded by Dr. W. W. H. Thackston,

*Resolved*, By the Virginia Society of Surgeon Dentists, that we believe the use of all PASTES and CEMENTS, of which MERCURY is a part, entirely unfit for, and highly objectionable, as fillings for carious teeth—that the use of them in dental practice is empirical AND IS HEREBY DECLARED TO BE MALPRACTICE.

*Resolved*, That while we reprobate the *use* of all such mercurial preparations, and will *execute our laws* with fidelity and promptness, we claim no authority over the *opinions* of our members, nor will we ever require of them other pledges than those which exist among *honorable men*, united for the purpose of improving and elevating a noble science.

Some debate arose upon the passage of these resolutions, whether the Society had power to expel its *members* for their opinions, or to convert its *executive officers* into father confessors to receive the confession of delinquents and grant absolution for offences. It was conceded by all that participatad, that the Society was not a court of conscience; that they had no right to demand any expression of opinions, or to require any pledge from them in relation to the use of mercurial *pastes* and *cements*; that the constitution prescribed the offence committed in their use, the Society having declared it *malpractice*, that the only course to pursue was to arraign an accused member, and upon conviction, execute the law. To expel for refusing to express an opinion, or to give a pledge, was making the refusal, *malpractice*, under the law, which was nonsensical and absurd. It was also contended, that to require action, under a threat, was a course of procedure to which no honorable man would submit. There were differences of opinion elicited during the debate, but the resolutions passed with but one dissenting voice—and as far as the use of the named materials were concerned, UNANIMOUSLY. It was ascertained that no member of this Society has used these preparations in his practice, unless in a few instances, as experiments, and that all condemn them as a substitute for gold. Drs. Sheppard, Thackston, M'Cabe, Hamlin and Wayt participated in the discussion.

On motion, *Ordered*, That the Secretary furnish a copy of the transactions of this meeting to the editors of "The American Journal of Dental Science," for publication.

On motion, *Ordered*, That the Secretary have published in pamphlet form, the transactions of the Society at this annual meeting, together with the charter, constitution and by-laws, and a full and accurate list of members, and that he transmit copies to each member of the Society and to such other persons as will tend to give them the greatest publicity.

On motion, *Ordered*, That the Secretary address a circular to each delinquent member of this Society calling upon each to pay up his arrears, or to appear at the next annual meeting to show cause why he should not be expelled for delinquency, and that the resolution fixing the period of future delinquencies be embraced in said circular.

On motion, *Resolved*, That the thanks of the Society be, and are hereby tendered to our President for his faithful, able and impartial discharge of his duties during the past and present meetings of the Society.

On motion, *Ordered*, That the thanks of this Society be, and are hereby tendered to the Secretary and Treasurer for their able, faithful and prompt discharge of their several official duties during the past year.

On motion of Dr. S. M. Sheppard, the Society adjourned to meet in the town of Petersburg on the second Wednesday in October, 1846.

S. LETHBRIDGE, D. D. S., PRESIDENT.

A true copy.

JAMES D. M'CABE, D. D. S., *Secretary*.

### APPENDIX.

The Virginia Society of Surgeon Dentists having heard that various individuals were in their professional cards publishing themselves as members of *this Society*, and thereby obtaining credit with the public, are induced to publish a full list of all its members. The gentlemen named below are the only persons *by them authorized* to practice Dental Surgery in Virginia:

Samuel Lethbridge, D. D. S., *President;* Richmond, Va.

John G. Wayt, D. D. S.*† *Vice-President;* Richmond, Va.

J. D. M'Cabe, D. D. S.*† *Sec'y;* Richmond. Retired.

S. M. Sheppard, D. D. S.,† *Treasurer;* Petersburg, Virginia.

W. W. H. Thackston, D. D. S.*† Farmville, Pr. Edward, Va.

John M'Connell, D. D. S.† Richmond, Virginia.

T. B. Hamlin, D. D. S.*† Wytheville, Wythe County, Va.

William N. M'Kenney, M. D.† Norfolk, Virginia.

John B. Garland, M. D., Fredericksburg, Virginia.

David M. Coulling, Hampton, Virginia.

Leroy Murrell, D. D. S.† Petersburg, Virginia.

J. W. Sheppard, D. D. S.† Petersburg, Virginia.

William C. Crump, M. D., Richmond, Virginia.

C. F. Martin, D. D. S.† Norfolk, Virginia.

John C. Burch. Now in the South.

John C. M'Cabe, D. D. S.*† Norfolk, Virginia.

Asa Pierce, D. D. S.† Sussex Court House, Virginia.

Benjamin W. Collier,*† Hick's Ford, (not practising now.)

George H. Leitch, Fredericksburg, Virginia.

Solomon Angel, D. D. S., Danville, Virginia.

John H. Blankenship, D. D. S., Charlottesville, Virginia.

Abner D. Lewis, D. D. S., Prince Edward County, Virginia.

### HONORARY MEMBERS.

C. A. Harris, A. M., M. D., D. D. S. Professor of Dental Physiology and Practical Dentistry, Baltimore College of Surgeon Dentists, Baltimore, Maryland.

William Tatum, M. D., Norfolk, Virginia.

While there may be many eminent practitioners in our State, not named above, these are all who have been accredited by the Virginia Society of Surgeon Dentists and who have received its Diploma. The action of the Society is directed to combine the talent and professional intelligence of the State in efforts to improve and elevate dental science, and they confidently expect that the honorable of the profession will unite with them to protect the public from the depredations and malpractice of the charlatan and empiric. If, however, the public will countenance unqualified and unauthor-

* Those marked thus have received the degree of Doctor of Dental Surgery in the Baltimore College of Dental Surgeons.

† Those marked thus are Fellows of the American Society of Dental Surgeons.

ized pretenders, to their own wrong, without demanding the *prima facie* evidence of a certificate or diploma from some reputable Society or College that they respect their profession and are qualified to practice it understandingly, then we must despair of accomplishing much which we now hope to perform. We, therefore, call upon the public to demand from all who offer their services in the treatment of dental diseases, a Diploma or Certificate, not from clergymen, doctors of medicine, or lawyers, but from the Dental College, or from some one respectable Society of Surgeon Dentists. If the individual is competent, he can easily obtain this testimonial, if he is not, he does not deserve your patronage.

Signed by order of the Society.

JAMES D. M'CABE, D. D. S., *Secretary.*

